# Removal of Chemical Oxygen Demand (COD) from Swine Farm Wastewater by *Corynebacterium xerosis* H1

**DOI:** 10.3390/microorganisms13071621

**Published:** 2025-07-09

**Authors:** Jingyi Zhang, Meng Liu, Heshi Tian, Lingcong Kong, Wenyan Yang, Lianyu Yang, Yunhang Gao

**Affiliations:** College of Veterinary Medicine, Jilin Agricultural University, Changchun 130118, China; zhangjingyi0309@126.com (J.Z.); liumeng4610@163.com (M.L.); 13614475772@163.com (H.T.); lingcong@jlau.edu.cn (L.K.); yangwy760913@163.com (W.Y.)

**Keywords:** swine wastewater, microbial biodegradation, FT-ICR MS, bioaugmentation, microbial community structure

## Abstract

Swine wastewater (SW) has a high chemical oxygen demand (COD) content and is difficult to degrade; an effective strategy to address this issue is through biodegradation, which poses negligible secondary pollution risks and ensures cost-efficiency. The objectives of this study were to isolate an effective COD-degrading strain of SW, characterize (at the molecular level) its transformation of SW, and apply it to practical production. A strain of *Corynebacterium xerosis* H1 was isolated and had a 27.93% ± 0.68% (mean ± SD) degradation rate of COD in SW. This strain precipitated growth in liquids, which has the advantage of not needing to be immobilized, unlike other wastewater-degrading bacteria. Based on analysis by Fourier-transform ion cyclotron resonance mass spectrometry (FT-ICR MS), this bacterium removed nitrogen-containing compounds in SW, with proteins and lipids decreasing from 41 to 10% and lignins increasing from 51 to 82%. Furthermore, the enhancement of the sequencing batch reactor (SBR) with strain H1 improved COD removal in effluent, with reductions in the fluorescence intensity of aromatic protein I, aromatic protein II, humic-like acids, and fulvic acid regions. In addition, based on 16S rRNA gene sequencing analysis, SBR_H1_ successfully colonized some H1 bacteria and had a higher abundance of functional microbiota than SBR_C_. This study confirms that *Corynebacterium xerosis* H1, as a carrier-free efficient strain, can be directly applied to swine wastewater treatment, reducing carrier costs and the risk of secondary pollution. The discovery of this strain enriches the microbial resource pool for SW COD degradation and provides a new scheme with both economic and environmental friendliness for large-scale treatment.

## 1. Introduction

In China in 2023, pork production was 57.94 million tons, and 726.62 million pigs were slaughtered, marking increases of 4.6 and 3.8%, respectively, over the previous year [1]. While meeting the growing human demand for meat, pig farming also produces a large amount of swine wastewater (SW) [2], approximately 160 million tons annually, which is a main source of water pollution in China [3,4].

Chemical oxygen demand (COD) is a crucial metric for assessing organic contamination in water [5]. Swine wastewater is a mixture of pig excrement, feed, wash water, and other pollutants, with much organic matter, resulting in high levels of COD indicators [6,7,8]. The high COD of SW is potentially problematic; if not properly treated and discharged, it may decrease dissolved oxygen concentrations in water, adversely affecting aquatic life and leading to discoloration and odor issues, with detrimental effects on the aquatic ecosystem and public health [9,10].

The biological method of treating SW is widely used, as it is inexpensive and causes no secondary pollution [11]. For example, *Bacillus subtilis* has been used as the target bacterium and sodium alginate–chitosan as a carrier, with immobilized bacterial particles prepared under optimal conditions significantly improving nitrogen removal from ammonia in SW [12]. In addition, SW has also been treated with a microalga–bacterium combination (*Chlorella vulgaris*-activated sludge [AS]); following co-cultivation, the elimination of NH_4_^+^-N and PO_4_^3−^-P increased by 53.84 and 43.52%, respectively [13]. However, because SW has a high concentration of complex organic contaminants, there are limited species of degrading bacteria for SW and even fewer for SW COD [14,15]. Most bacteria grow in suspension in liquid, and microbial immobilization technology is needed to treat wastewater. This technology immobilizes biocatalysts such as microorganisms onto specific media by physical or chemical means [16]. Immobilization can significantly enhance process stability, effectively resist environmental fluctuations, and ensure stable processing efficiency, as well as improving the biomass retention rate, elevating the biomass concentration of the treatment system, and accelerating the degradation of complex organic matter [17]. Immobilized carriers are the core of immobilization technology. In recent years, new immobilized carriers have emerged continuously, such as composite carriers, nanomaterials, metal–organic frameworks (MOFs), and biochar materials [18]. These carriers have great potential for enhancing microbial load, improving microbial stability and activity, and creating a better living environment for microorganisms, holding promise for the development of COD-degrading bacteria suitable for SW. However, microbial immobilization technology faces multiple challenges in environmental remediation applications: the high cost and frequent replacement of carriers; poor stability of carriers, which are prone to fragmentation and pose risks of secondary pollution; easy detachment of microorganisms; hindered substrate mass transfer; restricted cell activity, and thus narrow application scope of the technology [19]. Therefore, there is an urgent need to develop an efficient and suitable SW COD-degrading bacterium to better deal with the problem of SW treatment.

Fourier-transform ion cyclotron resonance mass spectrometry (FT-ICR MS), the highest resolution mass spectrometry, can accurately identify the molecular formula of compounds by combining the elemental composition of carbon, hydrogen, oxygen, nitrogen, and sulfur and provide comprehensive details regarding molecular-level characteristics of dissolved organic matter (DOM) molecules [20]. It has been widely used for wastewater treatment in recent years [21,22,23]. COD and DOM are core parameters for evaluating organic pollution in water environments, exhibiting both essential differences and dynamic coupling relationships. DOM refers to dissolved organic matter with a particle size < 0.45 μm. Studies have shown that DOM accounts for 78.1–86.5% of the total COD in wastewater, serving as a significant component of COD [24]. The content of DOM is positively correlated with COD values. Additionally, the composition and properties of DOM can affect the results of COD determination. In the research on COD removal mechanisms, as the main contributor to COD, DOM plays a key role in the complex relationship between the two. Therefore, analyzing the composition and changes of DOM is central to studying COD removal mechanisms. However, molecular-level studies on the transformation of DOM in SW before and after bacterial treatment are currently unclear.

The objective of this study was to isolate an effective strain of SW COD-degrading bacteria and characterize its transformation of SW at the molecular level using FT-ICR-MS. In addition, it was used in an enhanced SBR system and analyzed by three-dimensional fluorescence spectroscopy (3D-EEM) and 16S rRNA gene sequencing for the aqueous and sludge phases, respectively, to assess the removal performance and bioaugmentation potential. Notably, to the best of our knowledge, this is the first report of utilizing *Corynebacterium xerosis* for COD degradation in SW. This work enriches the resources of microbial strains for COD removal, provides insights into the mechanisms of biodegradation of COD, and is a guide for practical application in SW treatment processes.

## 2. Materials and Methods

### 2.1. Degradation Capacity

Several Erlenmeyer flasks were prepared, each filled with 100 mL of SW from the oxidation pond of a pig farm in Changchun City, collected in September at approximately 25 °C with a pH of 6.55 ± 0.06, and its detailed physicochemical properties are provided in Supplementary Table S2. The samples were collected on the same day, transported to the laboratory using a cold chain, and stored at 4 °C in a refrigerator for future use. The SW was autoclaved at 121 °C for 20 min, resulting in an initial COD concentration of 1276.96 ± 11.75 mg/L. A total of 20 bacterial strains were isolated, including 5 from the activated sludge of a municipal wastewater treatment plant in Changchun, 6 from the wastewater of a cattle farm in Changchun, 4 from the wastewater of a pig farm in Changchun, and 5 from a commercially available wastewater treatment bacterial agent product. During isolation, 50 mL of each environmental sample was added to a 250 mL sterilized Erlenmeyer flask containing glass beads, mixed with 50 mL of sterile water, and shaken at 30 °C and 130 rpm for 1 h to release microorganisms. The samples were serially diluted to 10^−3^, 10^−4^, and 10^−5^, spread on solid medium, and incubated at 37 °C for 24 h. Colonies with obvious growth advantages and unique morphology/color were selected for streak purification, and the purity was verified by microscopic observation until pure cultures were obtained. Various bacterial strains were inoculated into Tryptic Soy Broth (TSB), incubated at 30 °C and 130 revolutions/min for 24 h, and then centrifuged for 5 min at 5000 rpm. After washing bacteria with PBS three times, the concentration of the bacterial solution was measured with OD_600_ (UV/visible spectrophotometer MU701, Shimadzu, Kyoto, Japan) and the OD_600_ value was adjusted to 1.0. Then, 1 mL of bacterial suspension was added to each experimental Erlenmeyer flask, while the control group had no bacterial suspension added, with other conditions unchanged. Each group had 3 replicates, and all flasks were incubated at 30 °C and 130 r/min for 48 h. To determine COD content and calculate the degradation rate, samples were collected approximately 2–3 cm below the liquid surface. The strains with the greatest COD degradation capacity were compared.

### 2.2. Strain Identification

The isolated strains were subjected to DNA extraction using the Ezup Column Bacterial Genomic DNA Extraction Kit (Sangon Biotech, Shanghai, China), according to the manufacturer’s instructions. The strains were amplified by PCR, using DNA as a template and the 16S universal primers 27F and 1492R. The PCR reaction system consisted of 12.5 μL 2x Taq PCR Mix, 1 μL each of upstream and downstream primers, 2 μL of template, and 8.5 uL dd H_2_O. The PCR primers were forward: 5′-AGAGTTTGATCCTGGCTCAG-3′; and reverse: 5′-GGTTACCTTGTTACGACTT-3′. After verification by agarose gel electrophoresis, sequencing data were sent to Sangon Biotech (Shanghai) Co., Ltd. Sequences were repeatedly aligned with other selected sequences of strains of the same family in the GenBank database using ClustalW alignment. The sequencing results were compared to known strains on NCBI online, and the sequences of some strains with high homology were downloaded to construct a phylogenetic tree of strain H1, based on MEGA 7.0 software [25].

### 2.3. Strain Characterization

Strain H1 was characterized for colony morphology and Gram staining. Scanning electron microscopy (SEM) was performed at the Shanghai Gertron Testing and Technology Center on a Tescan Mira 3 XH, which has the following specifications: a secondary electron image resolution of 1 nm (at 30 kV) and 3.5 nm (at 1 kV), a magnification range from 1 to 1,000,000, and an accelerating voltage from 1 to 30 kV. Before being sent for testing, samples were subjected to bacterial culture, cleaning, dehydration, and drying, as described in Wang et al. [26].

### 2.4. Fourier-Transform Ion Cyclotron Resonance Mass Spectrometry (FT-ICR MS)

The molecular makeup of SW DOM, before and after treatment with strain H1, was analyzed with FT-ICR MS (SolariX 7.0 T, Bruker, Billerica, MA, USA). The algorithm’s mass accuracy is within ±1 ppm, and its signal-to-noise ratio is >10. Regions on the van Krevelen diagram correspond to the seven categories of compounds that constitute NOM: lipids, carbohydrates, unsaturated hydrocarbons, aliphatics/proteins, aromatic structures, structures like CRAMs (lignin/carboxylic-rich alicyclic molecules), and tannins. The calculation methods of molecular-level parameters follow Zhang et al. [27]. Data analysis, molecular formula matching, and sample pretreatment for FT-ICR MS analysis were performed at the Shanghai Jiaotong University analytical testing laboratory.

### 2.5. Sequencing Batch Reactor (SBR) Operation and Setup

The total effective volume of the SBR was 3.5 L. The feed water to the SBR was 1.25 L per cycle, and the ratio of feed water to drain water was 0.36; the dissolved oxygen content was maintained at 3–4.5 mg/L during the aeration process, and the stirrer rotated at a speed of 130 r/min. The activated sludge inoculated in the SBR came from the secondary sedimentation tank of a wastewater treatment plant in Changchun City, and the MLVSS was stable at 2.5 g/L. The influent was the same raw SW described in 2.1, which was collected from the oxidation pond of a pig farm in Changchun City in September, and stored at 4 °C after being transported via cold chain. After the two groups of SBR units were stabilized, parameters were adjusted consistently and set for the control group and the experimental group (SBR_C_ and SBR_H1_, respectively). Strain H1 was cultured and treated according to the method described in 2.1, and the OD_600_ value of the bacterial suspension was adjusted to 1.0. Then, 1% of the total effective volume of the SBR (3.5 L), i.e., 35 mL, was used as the inoculation volume, and this bacterial suspension was added to the SBR_H1_ reactor. The operation cycle was for 8 h, three times a day (water intake 20 min, mixing 50 min, aeration 240 min, sedimentation 120 min, drainage 10 min, and idle 40 min), controlled by a time controller. The test was run for 14 d. Two sets of influent, effluent, and activated sludge samples were collected in triplicate each day for subsequent measurements.

### 2.6. Three-Dimensional Fluorescence Spectroscopy (3D-EEM)

All samples were prepared as a solution with a TOC of 10 mg/L and filtered through a 0.45 μm filter membrane prior to 3D fluorescence detection. The Hitachi F7000 (Hitachi, Tokyo, Japan) was the scanning instrument used; the test environment was maintained at approximately 25 °C; the emission wavelength was Em: 250–550 nm; the occurrence scanning interval was 1 nm; excitation wavelength was Ex: 200–500 nm; the excitation light source was a 150 W xenon arc lamp; and the scanning speed was 12,000 nm/min. The width of the excitation and emission slit was 5 nm, and the PMT voltage was 700 V. EEM spectral data analysis and fluorescence response percentage calculation were conducted using the FRI technique (Pi, *n*, %) [28]. Fluorescence intensity data associated with all wavelengths in the EEM spectra were analyzed. Five emission zones were identified from the entire EEM spectrum, based on the fluorescence of the DOM components. Three-dimensional fluorescence spectral mapping was performed using Matlab 2018.

### 2.7. The 16S rRNA Gene Sequencing

Activated sludge samples from Days 1, 5, and 14 of operation of the two SBR units were collected, and 50 mL of thickened sludge was collected after centrifugation at 10,000 rpm for 5 min and stored at −80 °C. Lianchuan Biotechnology Co. was tasked with the extraction of total DNA, species annotation, diversity analysis, and 16S rRNA gene V3–V4 region sequencing for every sample. The primers used for 16S rRNA gene sequencing were 341F (5′-CCTACGGGNGGCWGCAG-3′) and 805R (5′-GACTACHVGGGTATCTAATCC-3′). For sequencing, the NovaSeq 6000 sequencing platform was employed, conducting paired-end sequencing with a configuration of 2 × 250 bp. The corresponding reagent used was the NovaSeq 6000 SP Reagent Kit (500 cycles). For sequencing data analysis, the bioinformatics tools used were FLASH for merging paired-end reads, fqtrim (v0.94) for quality filtering, Vsearch (v2.3.4) for filtering chimeric sequences, DADA2 for dereplication, QIIME2 (2023.2) for calculating diversity, BLAST+ (v2.12.0) for sequence alignment, and SILVA (release 138) for annotating feature sequences.

### 2.8. Conventional Index Analysis

The parameters to be tested during the test were COD, NH_4_^+^-N, TN, and TP, which were measured by a multi-parameter water quality meter (TR 6900). The instrument had a measurement error ≤±5% and repeatability ≤±5%. The potassium dichromate method was used to measure COD, whereas Nessler reagent spectrophotometry was used to measure NH_4_^+^-N. TN was determined by the persulfate oxidation method and TP by ammonium molybdate spectrophotometry. The above detection methods refer to the “Water and Wastewater Monitoring and Analysis Method” [29].

### 2.9. qPCR Validation

Activated sludge was collected from the 1st, 5th, and 14th days of the SBR_C_ and SBR_H1_ runs. Isolation and purification were performed using a soil DNA kit of total DNA (Omega Biotech, Doraville, GA, USA) via TB Green^®^ Premix Ex Taq™ II (Takara, Tokyo, Japan). Real-time quantitative PCR was performed with the kit, and the relative expression of H1 was normalized to 16S rRNA. The specific primer of strain H1 was synthesized by Comate Bioscience Co., Ltd. (Jilin, China), and the primer sequence was as follows: forward: 5′-CATGGGTAGCGAACAGGATTAG-3′; reverse: 5′-GTTAGCTACGGCACAGAAGTC-3′. The reaction procedure was 95 °C for 5 min, (95 °C for 10 s, 60 °C for 30 s, 40 cycles), 95 °C for 15 s, 60 °C for 60 s, 95 °C for 15 s, and 37 °C for 30 s. Three technical replicates were set for each set of experimental samples, the LightCycler^®^ 96 (Roche, Basel, Switzerland) was subjected to quantitative real-time PCR, and the relative expression level of H1 was determined according to Equation (2)^−(ΔΔCt)^ [30].

### 2.10. Viable Count of Corynebacterium xerosis

To evaluate the survival quantity of Corynebacterium xerosis in the sludge–water mixture of the control group (SBR_C_) and the experimental group (SBR_H1_) on the 1st, 5th, and 14th days of operation, potassium tellurite blood agar identification medium (Qingdao High-tech Industrial Park Haibo Biotechnology Co., Ltd., Qingdao, China) was used for viable count. This medium can specifically promote the growth of Corynebacterium xerosis and inhibit miscellaneous bacteria. The samples were diluted 10^3^ times, and then 100 μL of the diluted solution was spread on the culture plate. Three biological replicates were set for each group. After culturing at 30 °C for 48 h, the plates with a number of colonies ranging from 30 to 300 were selected, and the colonies were counted according to the characteristics of black colonies. The viable count was determined by the spread plate method, and the results were expressed as mean ± standard deviation (mean ± SD, CFU/mL). Single colonies were randomly selected and subjected to molecular identification according to the method described in Section 2.2 to verify the species specificity of the colonies.

### 2.11. Statistical Analysis

GraphPad Prism 8 was employed for data analysis and plotting. For comparisons among multiple groups, one-way analysis of variance (ANOVA) with Duncan’s multiple comparison test was performed, after verifying normality and homoscedasticity assumptions. Differences between two groups were evaluated using the unpaired *t*-test. Data were presented as mean ± standard deviation (mean ± SD).

## 3. Results and Discussion

### 3.1. COD Degradation Ability of Each Strain

**Figure 1:**
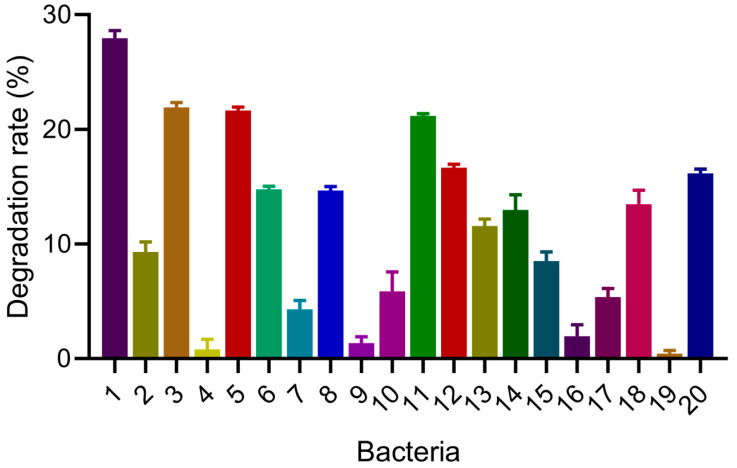
COD degradation rate of 20 strains of bacteria.

The COD degradation rate of SW of 20 strains of bacteria is shown (Figure 1); each strain had its own degradation ability, with strain No. 1, denoted “H1,” having the strongest degradation ability (27.93% ± 0.68%), which was significantly better than other strains (*p* < 0.001). The 20 strains of bacteria were preliminarily identified through 16S rRNA gene sequence analysis. The results showed that strain No. 1 was *Corynebacterium xerosis*. The identification results of the remaining strains were as follows: *Lysinibacillus alkaliphilus* (No. 2), *Acinetobacter* sp. (No. 3), *Comamonas kerstersii* (No. 4), *Lysinibacillus* spp. (No. 5, 6, 13), *Comamonas aquatica* (No. 8), *Bacillus subtilis* (No. 9, 10, 16, 18), *Stenotrophomonas maltophilia* (No. 11), *Rhodococcus* sp. (No. 12), *Serratia marcescens* (No. 14), *Acinetobacter* spp. (No. 7, 15), *Bacillus velezensis* (No. 17, 19), and *Bacillus licheniformis* (No. 20). In previous similar studies, a strain of *Bacillus velezensis* reduced COD in abattoir wastewater with an 11.80% degradation efficiency [31]. *Bacillus velezensis* was immobilized on polyvinyl alcohol (PVA) microspheres, with a 16.99% removal of COD from abattoir wastewater [32]. Based on previous studies, we inferred that strain H1 demonstrated strong potential for removing COD from farm wastewater with high concentrations, and therefore, it should be further investigated.

### 3.2. Identification and Characterization of Strain H1

**Figure 2:**
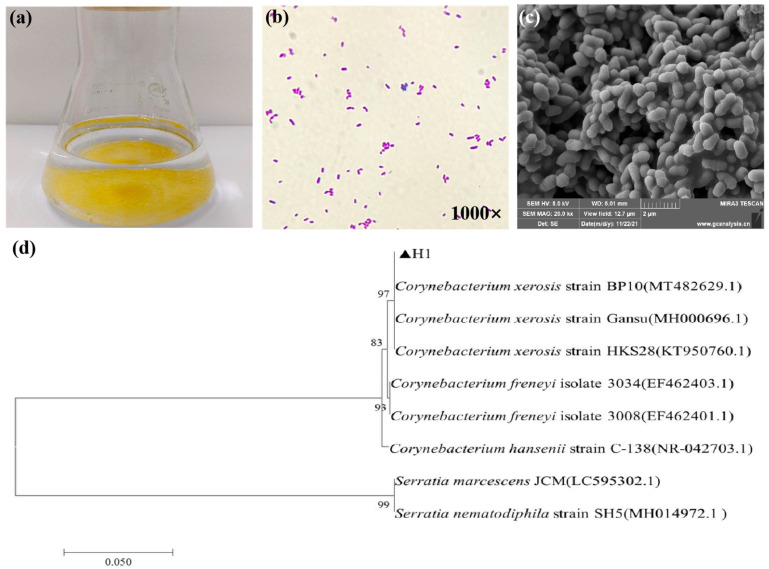
(**a**) Bacterial appearance, (**b**) Gram staining, (**c**) SEM image, and (**d**) phylogenetic tree analysis of strain H1 (the neighbor-joining method based on 1000 bootstraps was used).

Strain H1 grew precipitately in liquid, yielding a clear supernatant and a yellow granular precipitate (Figure 2a). After shaking, it settled quickly, conferring the advantage of not requiring immobilization in wastewater treatment. The strain was Gram-positive (Figure 2b) with short rods (Figure 2c), and the bacteria adhered to each other, forming large aggregates that promoted rapid sedimentation. The 16S rRNA gene fragment of strain H1 was 1371 bp (Figure 2d), and this sequence was submitted to GenBank (accession number PP474286). Using BLAST+ (v2.12.0) online comparison, gene sequences of standard strains with high homology to strain H1 were selected to construct a phylogenetic tree, with strain H1 most homologous to *Corynebacterium xerosis* strain BP10 (MT482629.1). Combined with the colony of strain H1 and its morphological, physiological, and biochemical characteristics, H1 was identified as *Corynebacterium xerosis*. As this strain has not been reported to degrade COD and no related pathogenicity was identified, it was necessary to conduct further studies [33].

### 3.3. DOM Molecular Transformation During SW Treatment by Strain H1

**Figure 3:**
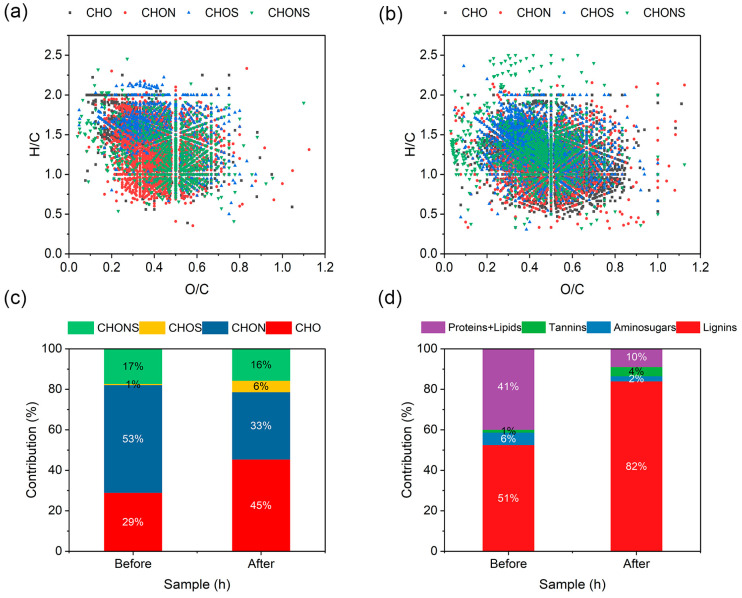
(**a**) van Krevelen diagram of original swine wastewater (SW); (**b**) van Krevelen diagram of strain H1-treated SW; (**c**) bar graph of major compounds in DOM samples; (**d**) bar graph of major organics in DOM samples.

FT-ICR-MS was used to analyze the molecular makeup of DOM in untreated and strain H1-treated SW. The evolutionary pattern of DOM’s molecular composition across the strain H1 treatment of SW was characterized by visualizing molecular structures using van Krevelen diagrams. Four elemental composition classes—CHO, CHON, CHOS, and CHONS—were identified in SW before and after treatment with strain H1, but the relative abundances of these classes varied significantly (Figure 3c). The proportion of compound CHON in the original SW after treatment with strain H1 was reduced from 53 to 33%; the proportion of compound CHONS was reduced from 17 to 16%; the proportion of compound CHO was increased from 29 to 45%; and the proportion of compound CHOS was increased from 1 to 6%. Therefore, some N-containing compounds were removed from SW after treatment with strain H1. The removed CHON-like substances were mainly distributed at O/C < 0.5, indicating that lipids (H/C = 1.5–2.0; O/C = 0–0.2) and proteins (H/C = 1.5–2.2; O/C = 0.2–0.52) were significantly reduced in SW after treatment with strain H1 (Figure 3a,b). Based on various H/C and O/C ratios in van Krevelen diagrams, four major types of organics, namely, aminosugars, proteins + lipids, tannins, and lignins, were derived (Figure 3d). After treatment with strain H1, there was a significant decrease in the proteins + lipids group of organic matter in SW, from 41 to 10%, and a significant increase in the lignins group of organic matter, from 51 to 82%. This trend aligns with previous findings: when SW was treated by various processes (coagulation–air flotation, anoxic/aerobic, and coagulation–sedimentation), the proportion of lipids and of proteins decreased from 34.24 and 63.26% to 4.27 and 17.22%, respectively, whereas the proportion of lignins increased from 32.32 to 75.20% [34]. Clearly, strain H1 affected the COD index by changing the compositional structure of proteins, lipids, and lignin-like organic matter in SW.

### 3.4. Biofortification Properties of Strain H1 in SBR

**Figure 4:**
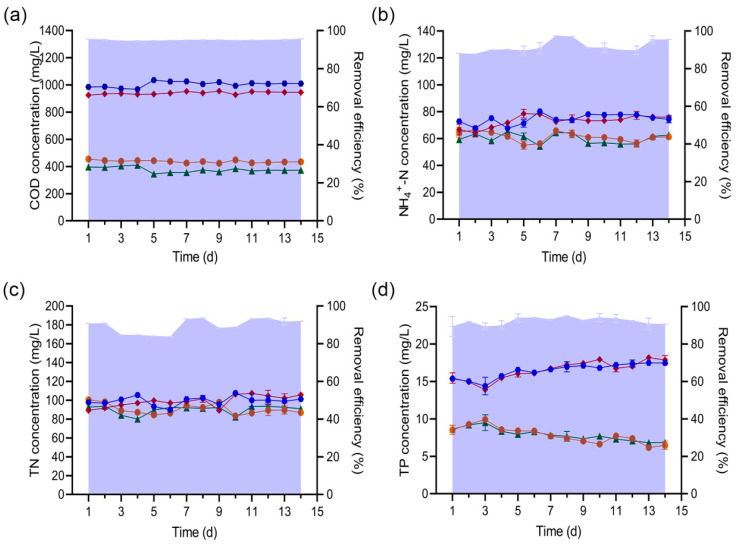
Changes in (**a**) COD, (**b**) NH_4_^+^-N, (**c**) TN, and (**d**) TP in SBR_C_ and SBR_H1_. Values are given as mean ± SD for replicates. Symbols: 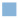 influent concentration, 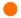 effluent concentration of SBR_C_, 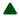 effluent concentration of SBR_H1_, 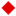 removal efficiency of SBR_C_, and 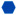 removal efficiency of SBR_H1_.

The control SBR_C_ and the test group SBR_H1_ were run for 14 d, according to the conditions set in the Materials and Methods. Water samples were gathered daily from the inflow and the outlet to determine concentrations of COD, NH_4_^+^-N, TN, and TP and to calculate removal rates. After strain H1 was added, the device SBRH1’s ability to remove COD was generally improved (Figure 4a). We performed an independent-samples Student’s *t*-test on the removal rate data of the two groups, and the difference in COD removal efficiency between SBR_H1_ and SBR_C_ was significant (*p* < 0.05), indicating that the COD removal efficiency of SBR_H1_ was significantly improved. Removal was best on Day 5, with the removal rate being approximately 7.36% higher compared to the SBR_C_ group (*p* < 0.05); after the 5th day, although the COD degradation rate of SBR_H1_ showed a tendency to decrease slightly and gradually stabilized, the difference in COD removal efficiency between SBR_H1_ and SBR_C_ remained statistically significant throughout the entire 14-day operating cycle (*p* < 0.05); that is, the COD removal efficiency of SBR_H1_ remained superior to that of the SBR_C_ group. COD removal ranged from approximately 66–68% for SBR_C_ to approximately 69–74% for SBR_H1_. The removal efficiency of COD by SBR_H1_ was significantly improved on the first day of strain H1 dosing, which is not common in bioaugmentation tests of SBR. For example, strain *P. stutzeri* XL-2 was used to enhance the denitrification performance of SBR, and the denitrification effect began to appear after ~8 d of bacterial dosing [35]. When *Pseudomonas aeruginosa* LX was immobilized to enhance the removal of ammonia and Cr(VI) from wastewater by SBR, effects began to appear after 3 d [36]. Furthermore, when *Pseudomonas* JMSTP was used to enhance SBR to treat simulated municipal wastewater, on the 10th day of inoculation with bacteria and after secondary inoculation, a stable TN removal effect began to appear [37].

The elimination of NH_4_^+^-N, TN, and TP did not differ significantly between the two sets of devices (*p* > 0.05), with removal rates of both groups in the ranges of approximately 46–57% for NH_4_^+^-N (Figure 4b), approximately 44–55% for TN (Figure 4c), and approximately 55–74% for TP (Figure 4d). Detailed numerical data for these removal rates are presented in Table S3 (Supplementary Materials). The change in outlet water was caused by the floating up and down of the inlet water. In summary, the addition of strain H1 increased SBR’s COD degradation rate without negatively affecting other end points. From Day 5, the effluent index of SBR_H1_ reached Emission Standards for Pollutants from Livestock and Poultry Farming (GB/T18596-2001) [38].

It is apparent that strain H1 has strong environmental adaptability, can function efficiently in a short interval, does not require immobilization or repeated inoculation, and has good potential for large-scale application. From the perspective of practical application, strain H1 is directly applied to sewage, avoiding the obstruction of material transfer caused by immobilized carriers, and can readily contact organic pollutants in SW, ensuring the efficient degradation of COD under large-scale conditions. Furthermore, the strain does not need carrier selection and immobilization reaction in immobilized bacteria technology, reducing the technical threshold and improving the function and stability of the process. Regarding costs, immobilized bacteria technology requires substantial investment in the procurement of immobilized materials and equipment, whereas strain H1 does not need to be immobilized, avoiding these costs. In addition, its stable activity ensures sustained and efficient treatment effects without additional strengthening measures, which not only reduces the procurement and replacement costs of immobilized carriers, but also reduces long-term operating costs, and avoids the risk of secondary pollution. Therefore, it has several benefits at both economic and environmental levels. However, this study has limitations in that SW samples were not collected across seasons, and long-term stability, large-scale feasibility, along with potential regulatory factors were not evaluated. These gaps may affect the generalizability of COD removal efficiency results across seasons and hinder practical implementation. Future research should assess the strain’s performance in multiple seasons, integrate feed formulation data, and address these unaddressed aspects to validate its application stability and feasibility.

### 3.5. Spectral Characterization of DOM in Swine Wastewater Treatment Processes

**Figure 5:**
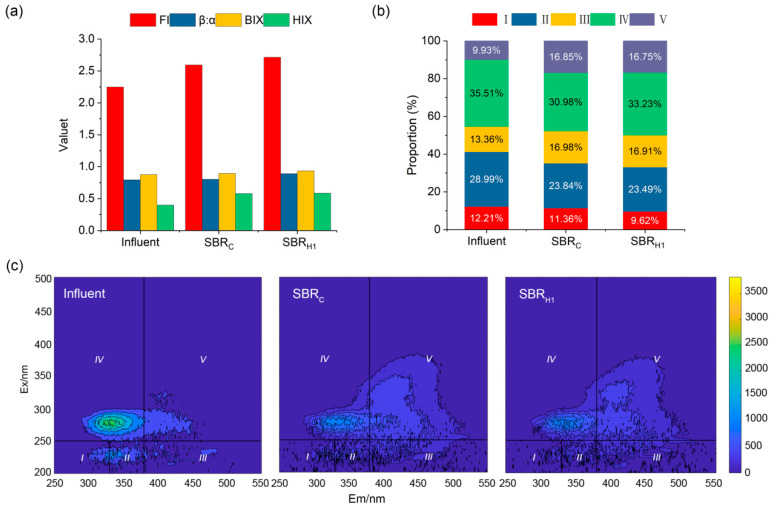
(**a**) Trends of FI, β:α, BIX, and HIX; (**b**) changes in the ratio of the five fluorescent components; (**c**) 3D-EEM spectra of the DOM of the inlet water, the SBR_C_ effluent on Day 5, and the SBR_H1_ effluent on Day 5. (I: aromatic protein I; II: aromatic protein II; III: fulvic acid analogs; IV: soluble microbial products; and V: humic acid analogs).

To characterize how bioaugmentation affected the SBR units’ effluent composition, three-dimensional fluorescence spectroscopy (3D-EEM) was performed on influent and effluent water from Day 5 of the two sets of units, SBR_C_ and SBR_H1_, based on the effluent index data. FI (fluorescence index), β:α (freshness index), BIX (biological index), and HXI (humification index) had the same outcomes, with SBR_C_ higher than influent and SBR_H1_ higher than SBR_C_ (Figure 5a). The influent FI index was >1.8, indicating that the DOM in the water body came from microbial degradation and was mainly related to anthropogenic organic pollutant sources. A higher FI index indicated higher bioavailability, whereas a higher β:α index indicated more newly produced DOM [39]. The BIX indexes were all > 0.8, indicating that DOM mainly came from biological activities within the water body; the autochthonous source was obvious, and the higher BIX indexes indicated that the self-purification ability and biological activity of the water body ecosystem increased [40]. The HIX metrics were all <4, indicating that the DOM was less humified and dominated by fresher, biologically sourced organic matter, and higher HIX metrics indicated higher system stability [41].

Five excitation emission regions were identified from the three-dimensional fluorescence spectrum: region I (Ex/Em = 200~250/280~330 nm) for aromatic protein I, region II (Ex/Em = 200~250 nm/330~380 nm) for aromatic protein II, region III (Ex/Em = 200~250/380~550 nm) for fulvic acid, region IV (Ex/Em = 250~450 nm/280~380 nm) for microbial by-products, and region V (Ex/Em = 250~450 nm/380~550 nm) for humic-like acids [42]. Both SBR_C_ and SBR_H1_ influents had significant fluorescence intensity, mainly in region IV and region II with 35.51 and 28.99% of regional integral, followed by region III, region I, and region V with 13.36, 12.21, and 9.93% of regional integral (Figure 5b,c). The SBR_C_ and SBR_H1_ influent was SW, and based on spectral results, wastewater contained a large amount of organic matter such as proteins, cellulose, hemicellulose, and lignin, accounting for the high COD content. Regions I, II, and IV had decreased fluorescence intensity of the wastewater treated by the SBR_C_ unit; correspondingly, the percentage of regional integral dropped from 12.21, 28.99, and 35.51% to 11.36, 23.84, and 30.98%. Meanwhile, regions III and V had increased fluorescence intensity, and the percentage of regional integral went from 13.36 and 9.93% to 16.98 and 16.85%. According to Wang et al. [43], these outcomes demonstrated that the device’s microbes quickly changed the readily broken down protein materials and decomposition of organic matter to form simple compounds, whereas products of microbial life activities were synthesized into humus-like and fulvic acid-like substances. After the wastewater enters the SBR_C_ unit, the microorganisms start working with their strong decomposition capabilities. In regions I and II, complex organic matter such as microbial by-products are disassembled, and small molecular substances containing carbon, nitrogen, phosphorus, etc., are released in region IV. At the same time, studies in soil science and other fields have shown that these small molecules will be used in the process of microbial metabolism to synthesize humic acids in region V through enzymatic reactions. During this period, the high expression of related synthetase genes in microbial cells drove carbon elements to construct polycyclic aromatic hydrocarbons and functional group structures unique to humic acids. These activities of microorganisms directly lead to a significant increase in regional V fluorescence intensity, reflecting that the composition structure of organic matter gradually changed to humus-like substances during the treatment process of the SBR_C_ device.

The fluorescence intensity of wastewater treated by SBR_H1_ decreased compared to the SBR_C_ group in regions I, II, III, and V. Among them, the decrease was more obvious in regions I and II, and the percentage of regional integrals decreased from 11.36 and 23.84% to 9.62 and 23.49%, which corresponded to the results of FT-ICR-MS. The fluorescence intensity of regions III and V somewhat decreased, and the percentage of regional integral decreased from 16.98 and 16.85% to 16.91 and 16.75%, indicating a decrease in the content of difficult-to-degrade organic matter [44].

The inclusion of strain H1 not only assisted the SBR device to more effectively remove various types of organic matter but also improved bioavailability, biological activity, and system stability.

### 3.6. Changes in Microbial Community Structure

Mud–water mixtures of the two systems, SBR_C_ and SBR_H1_, from Days 1, 5, and 14 were subjected to 16S rRNA gene sequencing analysis. To ensure the reliability of subsequent microbial community analysis, alpha diversity assessment was performed on the sequencing data by plotting Shannon curves (Figure S1). As shown in the figure, most curves tended to level off, indicating that with the increase in sequencing sequence numbers, the detected species diversity in samples no longer rose significantly. This suggests the current sequencing depth sufficiently reflects the true species diversity of samples, meeting the requirements for subsequent community structure analysis.

**Figure 6:**
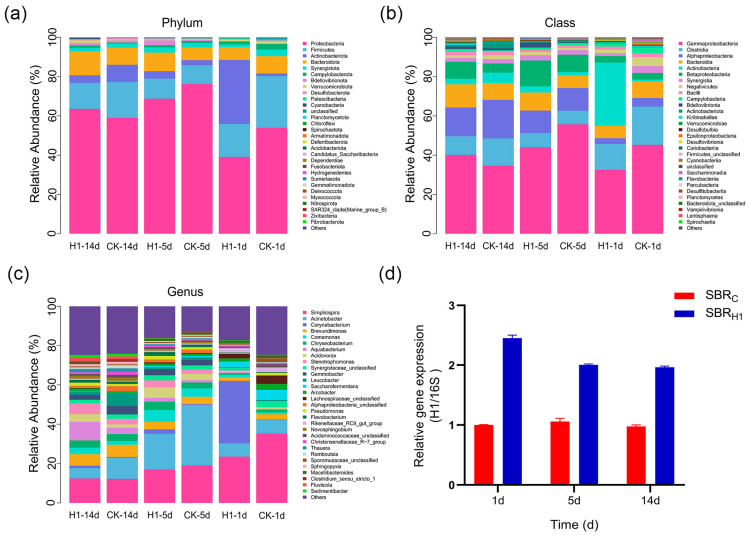
Differences in the relative abundance of bacterial communities for the activated sludge in the two systems at the (**a**) phylum, (**b**) class, and (**c**) genus levels, and (**d**) the relative gene expression at different time points.

The distribution of the top 30 colonies at the phylum, order, and genus levels for each sample is presented in Figure 6. Major microbiota in SBR_C_ and SBR_H1_ were the same; however, there were differences in the species’ relative abundances. At the level of phylum, *Proteobacteria*, *Firmicutes*, *Actinobacteriota*, and *Bacteroidetes* were the main dominant phyla in all samples, with *Proteobacteria* accounting for the largest share in all samples. On day 14, it accounted for 59.05% in the SBR_C_ sample and 63.61% in the SBR_H1_ sample (Figure 6a), in agreement with many previous reports [45]. Microorganisms in SBR_H1_ changed drastically after the addition of H1 bacteria on Day 1. The relative abundance of *Actinobacteriota* increased from 1.25% to 32.56%, whereas the relative abundances of all other prominent phyla decreased; the proportionate abundance of *Actinobacteria* decreased to 3.77%, and the abundance of all other dominant phyla increased in SBR_H1_ on Day 5 of systematic SBR_H1_; from an overall perspective, the abundance of *Actinobacteria* became increasingly lower with time and was finally lower than that of SBR_C_. *Proteobacteria*, *Bacteroidetes*, *Campylobacterota*, *Verrucomicrobia*, and *Patescibacteria* became increasingly abundant with time and eventually higher than SBR_C_. Both *Proteobacteria* and *Bacteroidetes* are considered important phyla for the biodegradation of organic matter in conventional activated sludge treatment systems. *Proteobacteria* had a key role in promoting sludge granulation and growth and an important role in COD and N removal, whereas *Verrucomicrobia* degraded dyes in anthraquinone dye wastewater [46,47,48,49].

At the class level, *Gammaproteobacteria*, *Clostridia*, *Alphaproteobacteria*, and *Bacteroidia* were the primary classes that dominated in each sample (Figure 6b), with *Gammaproteobacteria* accounting for the largest proportion of all samples. On day 14, it accounted for 34.69% in the SBR_C_ sample and 40.17% in the SBR_H1_ sample. The relative abundance of *Actinobacteria* in SBR_H1_ increased from 0.84% to 32.29% after the addition of H1 bacteria on Day 1, and the relative abundance of all other dominant classes decreased. Furthermore, the relative abundance of *Actinobacteria* in SBR_H1_ decreased drastically to 3.14% on Day 5, and the abundance of all other dominant classes, except *Clostridia*, increased. In addition, from an overall perspective, the abundance of *Actinobacteria* in SBR_H1_ became lower and lower than that of SBR_C_, whereas the abundance of *Gammaproteobacteria* and *Bacteroidia* increased with time and finally exceeded that of SBR_C_. Gammaproteobacteria had significant impacts on wastewater treatment by promoting COD degradation [48].

At the genus level, *Simplicispira* and *Acinetobacter* were the main dominant genera in all samples (Figure 6c). The relative abundance of *Corynebacterium* in the SBR_H1_ device increased from 0.31% to 31.81% on Day 1 due to the addition of dried *Corynebacterium* in SBR_H1_ on Day 1. The abundance of *Corynebacterium*, *Comamonas*, *Chryseobacterium*, *Acidovorax*, and *Stenotrophomonas* in SBR_H1_ was higher than that in SBR_C_ on Day 5; overall, *Corynebacterium* in SBR_H1_ became less and less abundant over time but was always higher than that in SBR_C_, whereas *Aquabacterium*, *Acidovorax*, and *Stenotrophomonas* became increasingly abundant over time and eventually higher than those in SBR_C_. Among them, *Acidovorax* and *Aquabacterium* are two typical NDFO bacteria that can oxidize ferrous iron to trivalent iron through a nitrate-dependent ferrous iron oxidation process with the simultaneous reduction of nitrate [50]. Biochemical reactions during this oxidation process may promote the decomposition or transformation of organic matter, thereby reducing COD. Alternatively, they can also work in concert with other microbes, promoting the decomposition of organic materials and removing nitrogen. Various potential denitrifying strains were also observed in the SBR_C_ and SBR_H1_ systems, such as *Acinetobacter*, *Simplicispira*, and *Comamonas* [35,51], and these genera may have some ability to remove Cr(VI) [52].

To investigate the colonization of strain H1 in the SBR unit, activated sludge samples were collected from the SBR_C_ group and the SBR_H1_ group on Days 1, 5, and 14, and the number of reactive strain H1 in the SBR was detected by qPCR (using H1-specific primers). There was a significant increase in the number of strain H1 in the SBR_H1_ group on Days 1, 5, and 14, and the number of strain H1 gradually stabilized with the extension of the SBR operation time (Figure 6d).

In this study, the survival numbers of *Corynebacterium xerosis* in the sludge samples of the SBR_C_ group and the SBR_H1_ group were simultaneously determined on the 1st, 5th, and 14th days of operation (Figure S2). At each time point, no colonies of *Corynebacterium xerosis* grew on the culture plates of the SBR_C_ group. Considering the experimental method and the characteristics of the culture medium, it was determined that *Corynebacterium xerosis* was absent in this group. In the SBR_H1_ group, the viable count on the 1st day was (8.43 ± 1.12) × 10^5^ CFU/mL, which decreased to (4.7 ± 0.6) × 10^5^ CFU/mL on the 5th day and reached (3.97 ± 0.62) × 10^5^ CFU/mL on the 14th day. The number of colonies was within the effective counting range of 30 to 300. All the colonies were black, which was consistent with the typical morphology of *Corynebacterium xerosis* on potassium tellurite blood agar medium. The species was further confirmed by molecular biological identification. In addition, the results of viable count and qPCR detection indicated that *Corynebacterium xerosis* achieved continuous colonization and maintained its activity in the SBR system.

Based on the above results, the initial stage of the SBR_H1_ system, due to a large number of H1 bacteria injected into the stable operation of the device, altered the structure of the original microbiota of the system due to the introduction of foreign microorganisms; in the middle stage of the SBR_H1_ system, the structure of the microbiota was restored, and the abundance of some of the dominant microbiota was higher than that of the SBR_C_; and finally, the stabilization phase of the SBR_H1_ system successfully colonized some H1 bacteria (without a need for immobilization) and therefore increased the proportion of other microbiota with functions such as organic matter degradation and nitrogen removal. These results corresponded to effluent results, as the addition of H1 bacteria changed the bacterial community structure of the SBR_H1_ system and increased the abundance of functional bacterial microbiota, promoting COD degradation.

## 4. Conclusions

The COD degradation of SW by strain H1 was 27.93 ± 2.33% and was identified as *Corynebacterium xerosis*. Strain H1 removed a certain amount of N substances in the compounds contained in SW and affected the COD index by changing the structure of proteins and lipids and lignin-like organic matter. In addition, strain H1 helped the SBR to increase the COD degradation rate without negatively affecting other indicators and reduced the content of aromatic protein I, aromatic protein II, fulvic acid, and humic acid-like organic matter in the SBR effluent. Strain H1 not only could be successfully colonized in the SBR device without immobilization but also increased the abundance of other functional microbiota in the device.

In summary, strain H1 can be considered a promising candidate strain for SW treatment for the removal of SW COD in order to improve the treatment efficiency of SW. Notably, translating this bioaugmentation strategy to industrial-scale application may face technical challenges. Furthermore, the potential pathogenicity of *Corynebacterium xerosis* strain H1 used in this study has not been systematically investigated, and further evaluation of its biosafety in practical applications is needed. Future research could focus on these key issues to promote the engineering application of this technology and verify its safety.

## Data Availability

The original contributions presented in this study are included in the article/Supplementary Materials. Further inquiries can be directed to the corresponding author.

## Supplementary Materials

The following supporting information can be downloaded at: https://www.mdpi.com/article/10.3390/microorganisms13071621/s1, Figure S1: Schematic diagram of dilution curve resultst; Figure S2: Colony situations of *Corynebacterium xerosis* in the sludge from the SBR_C_ and SBR_H1_ devices at different operation times on the potassium tellurite blood agar identification medium (with a dilution factor of 10^3^); Table S1: Abbreviation List; Table S2: Physicochemical Characteristics of Raw Swine Wastewater (SW); Table S3: Table of Pollutant Removal Efficiencies of SBRc and SBR_H1_ Systems under Different Operating Times.
