# Supplementary material for: Removal of Chemical Oxygen Demand (COD) from Swine Farm Wastewater by Corynebacterium xerosis H1
Source: Microorganisms. 2025 Jul 9;13(7):1621. doi: 10.3390/microorganisms13071621 (PMC12298782; doi:10.3390/microorganisms13071621)
Supplement: Supplementary file 1 [file microorganisms-13-01621-s001.zip › microorganisms-3667478-supplementary.pdf]

Supplementary Materials

Removal of Chemical Oxygen Demand (COD) from Swine Farm Wastewater by *Corynebacterium xerosis* H1

Jingyi Zhang, Meng Liu, Heshi Tian, Lingcong Kong, Wenyan Yang, Lianyu Yang * and Yunhang Gao *

College of Veterinary Medicine, Jilin Agricultural University, Changchun 130118, China
* Correspondence: yangly@jlau.edu.cn (L.Y.); gaoyunhang@163.com (Y.G.)

Table S1. Abbreviation List.

Abbreviation	Full Form
SW	Swine wastewater
COD	Chemical oxygen demand
FT-ICR MS	Fourier transform ion cyclotron resonance mass spectrometry
DOM	Dissolved organic matter
SBR	Sequencing batch reactors
SEM	Scanning electron microscope
3D-EEM	Three-dimensional fluorescence spectroscopy

Table S2. Physicochemical Characteristics of Raw Swine Wastewater (SW).

Index	Value	Unit
COD	1254.33±2.52	mg/L
NH_4^+-N	123.38±1.13	mg/L
TP	22.11±0.37	mg/L
TN	183.11±1.53	mg/L
pH	6.55±0.06	/

Table S3. Table of Pollutant Removal Efficiencies of SBRc and SBRh1 Systems under Different Operating Times.

Time (d)	COD Removal Efficiency (%)		NH_4^+-N Removal Efficiency (%)		TN Removal Efficiency (%)		TP Removal Efficiency (%)	
	SBR_C	SBR_{H1}	SBR_C	SBR_{H1}	SBR_C	SBR_{H1}	SBR_C	SBR_{H1}
1	66.06 ±0.11	70.36 ±0.12	47.63 ±2.93	52.01 ±0.24	44.61 ±0.81	48.85 ±0.73	61.87 ±2.57	61.46 ±0.48
2	66.75 ±0.37	70.53 ±0.37	45.92 ±2.13	48.35 ±1.11	45.89 ±1.16	48.45 ±1.00	59.78 ±0.36	60.10 ±0.31
3	66.92 ±0.10	69.51 ±0.11	48.76 ±0.73	53.66 ±1.58	47.52 ±1.07	50.36 ±1.80	55.60 ±0.54	57.51 ±4.72
4	66.52 ±0.36	69.14 ±0.38	51.20 ±0.81	48.34 ±1.31	48.53 ±1.36	52.79 ±0.98	61.97 ±0.58	62.92 ±0.49
5	66.60 ±0.21	73.97 ±0.17	56.11 ±2.38	50.91 ±2.02	49.82 ±0.94	46.70 ±0.50	64.25 ±1.78	66.25 ±1.24
6	67.11 ±0.19	73.25 ±0.21	55.82 ±1.58	57.33 ±0.96	48.54 ±0.72	44.89 ±1.23	64.43 ±1.56	64.80 ±0.74
7	68.04 ±0.22	73.28 ±0.22	51.84 ±1.11	52.88 ±0.73	49.33 ±0.69	50.59 ±1.10	66.93 ±0.48	66.44 ±1.65
8	67.23	71.87	53.31	52.69	50.78	51.24	68.95	67.83

	±0.23	±0.24	±2.06	±0.33	±1.76	±0.68	±0.65	±2.63
9	68.23	72.93	52.33	55.78	44.60	47.83	69.72	68.45
	±0.14	±0.15	±1.07	±1.28	±0.72	±0.76	±0.96	±1.31
10	66.27	71.02	52.35	55.50	53.16	53.83	71.81	67.27
	±0.10	±0.14	±0.31	±0.25	±0.84	±0.60	±1.47	±1.81
11	67.95	72.38	52.87	55.60	53.69	49.97	67.04	68.86
	±0.20	±0.19	±1.47	±1.83	±0.99	±3.32	±1.93	±0.84
12	67.74	72.02	55.18	55.47	52.28	50.06	68.07	69.53
	±0.32	±0.33	±1.98	±0.78	±2.88	±2.11	±1.78	±1.95
13	67.60	72.19	54.39	53.91	51.07	49.45	72.80	69.97
	±0.05	±0.03	±1.29	±1.34	±2.23	±1.68	±0.73	±1.76
14	67.56	72.15	54.18	53.12	52.82	50.64	71.47	69.84
	±0.08	±0.07	±1.16	±1.92	±1.32	±1.37	±2.16	±1.59

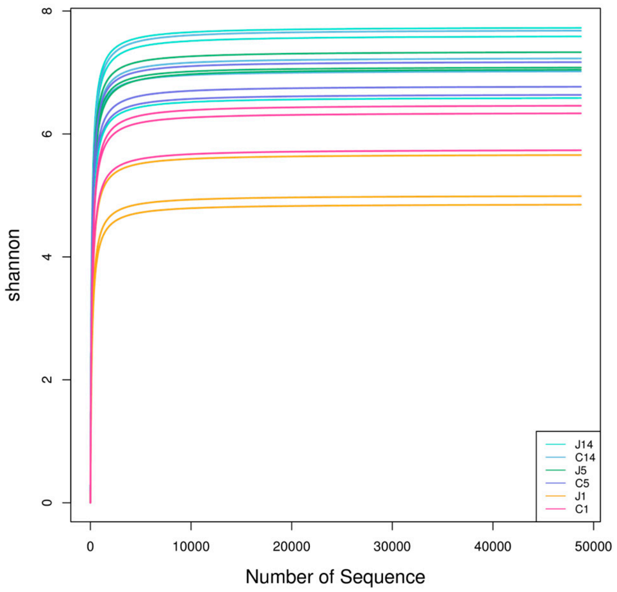

Figure S1. Schematic diagram of dilution curve results.

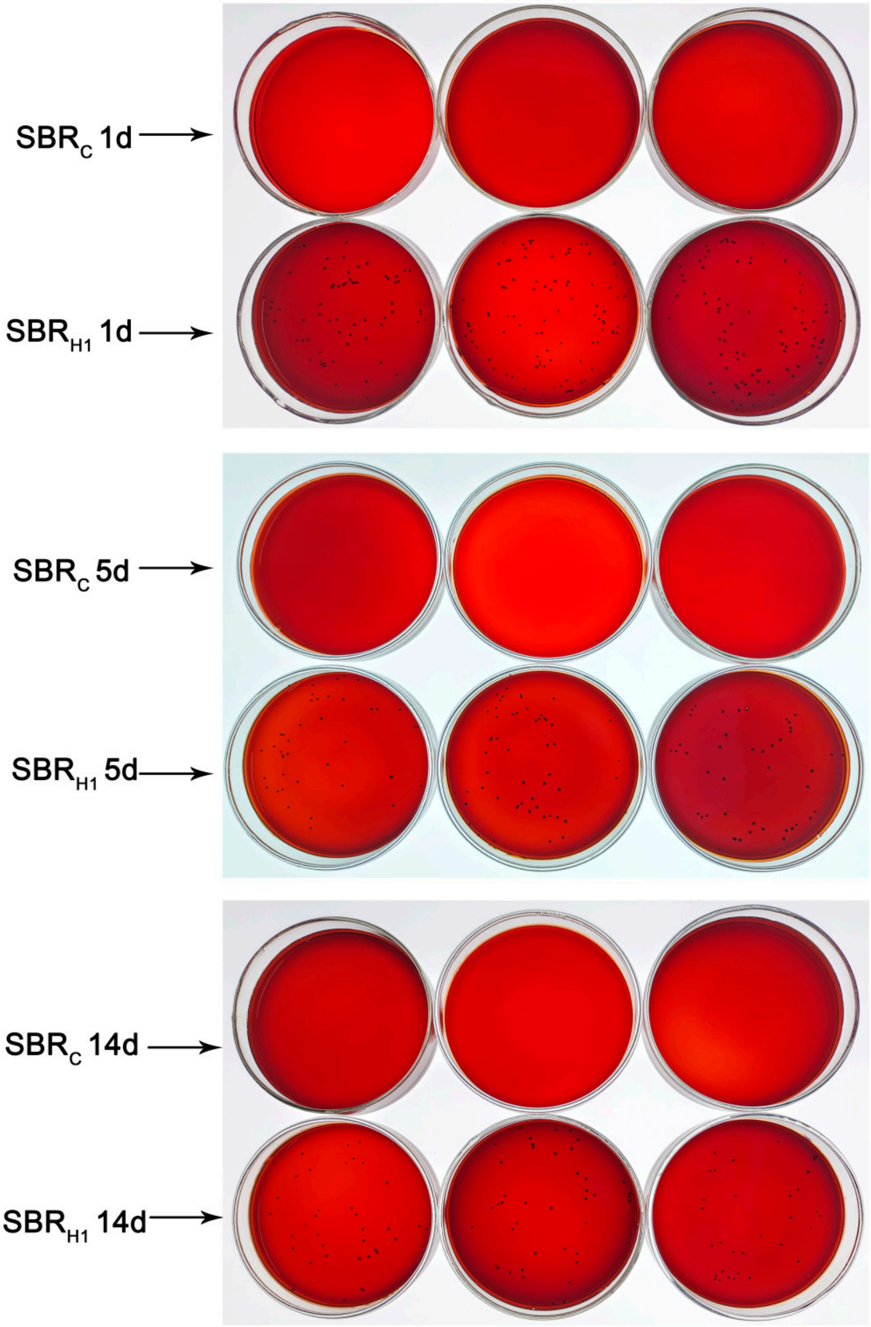

Figure S2. Colony situations of *Corynebacterium xerosis* in the sludge from the SBR$_C$ and SBR$_{H1}$ devices at different operation times on the potassium tellurite blood agar identification medium (with a dilution factor of 10^3).
